# Best Management Strategies for Sustainable Giant Clam Fishery in French Polynesia Islands: Answers from a Spatial Modeling Approach

**DOI:** 10.1371/journal.pone.0064641

**Published:** 2013-05-28

**Authors:** Simon Van Wynsberge, Serge Andréfouët, Antoine Gilbert, Arsène Stein, Georges Remoissenet

**Affiliations:** 1 UR-CoRéUs, Institut de Recherche pour le Développement, Nouméa, New-Caledonia; 2 Ginger Soproner, Nouméa, New-Caledonia; 3 Direction des Ressources Marines, Papeete (Fare Ute), French Polynesia; Leibniz Center for Tropical Marine Ecology, Germany

## Abstract

The giant clam *Tridacna maxima* has been largely overexploited in many tropical regions over the past decades, and was therefore listed in appendix II of the Convention of International Trade in Endangered Species (CITES) in 1985. In French Polynesia, several atolls and islands harbor the world’s highest stocks of giant clams in very shallow and accessible areas, which are therefore highly vulnerable to fishing pressure. The local fishery authority (i.e., Direction des Resources Marines or “DRM”) implemented several management schemes in 2002 to control and regulate fishing pressure. However, for further decisions DRM was missing a sensitivity analysis on the effectiveness of the possible management actions. Here, we report on the use of a deterministic Viable Population Analysis (VPA) and spatially-explicit age-based population model that simulated the 30-year trajectory of a *Tridacna maxima* stock under different management approaches. Specifically, given various scenarios of intra-island larval dispersal, we tested which of No-take-Areas (NTAs), rotational closures, size limits, quotas, and restocking schemes would lead to the highest future stocks in Tubuai and Raivavae, two exploited islands of the Austral archipelago. For both islands, stock abundances were estimated in 2004/2010 and 2005/2010 respectively, and natural mortalities were assessed previously only in Tubuai. When compared to field data, the model successfully predicted the 2010 stocks for Tubuai, but proved to be less reliable for Raivavae, where natural mortality rates may well be different from those on Tubuai. For Tubuai, the spatial model suggested that reducing fishing effort (through fixed quotas) and banning fishing below the 12 cm size limit (as currently implemented) were the most effective management actions to sustain *T. maxima* populations into the future. Implementing NTAs was of poor effectiveness. NTAs increased giant clam stock inside the protected area, but also increased overfishing in the neighboring areas, and were ineffective overall.

## Introduction

In 2009, around 80 million tons of marine resources (e.g., fish, and invertebrates such as clams, sea cucumbers) were captured worldwide [1]. The proportions of overexploited and depleted stocks were 28 and 3 percent, respectively, in 2008 [1]. These estimates have raised significant concerns [2], [3]. Keeping harvests within sustainable limits is critical to maintain food security [4], large stocks and high profits [5]. To this end, efficient fishery management is needed, but it is impaired by complex interactions between local, national and international regulations, with often competing concerns, and equally complex networks of stakeholders with various interests. In practice, the joint implementation of both top-down governmental and bottom-up community-based actions may contribute to the efficient management of fishery resources.

One relevant tropical example is the fishery for the giant clam *Tridacna maxima.* This species has been largely overexploited in many tropical regions over the past decades [6]. As a result, it was listed in Appendix II of the Convention on International Trade in Endangered Species (CITES) in 1985. This means that all Tridacnidae in international trade are subject to strict regulations and monitoring [7]. In some of French Polynesia’s lagoons, giant clam densities can be extremely high [8], [9], with stocks having been qualified as “extraordinary” for some atolls of the Eastern Tuamotu. For instance, 23.6±5.3 million clams (mean ±95% confidence interval) were recorded in the 4.05 km^2^ Fangatau lagoon [10] and 88.6±10.5 million clams in the 11.46 km^2^ Tatakoto lagoon [9]. Despite harbouring these sizeable stocks, increasing clam meat export towards the main market of Tahiti island, together with massive natural mortality events [11], [12], [13], have raised concern for the long-term sustainability of giant clam fisheries. As a consequence, the French Polynesia fishery authority (i.e., Direction des Resources Marines or “DRM”) has established several management actions to control and regulate fishing pressure.

First, in 1988, the following measures were implemented territory-wide: 1) a minimum body size catch-limit at 12 cm; and 2) the monitoring of local and international exports from remote islands towards and through the Tahiti market. The second step has been to implement additional actions that are more local and specific in scope. These actions included: 1) the evaluation of giant clam stock abundance for two Austral Archipelago islands and five Tuamotu Archipelago atolls [14], [9]; 2) an assessment of giant clam population dynamics (mortality and growth) and fishing pressure for two atolls (Tatakoto, Fangatau) and one island (Tubuai) [15]; and 3) the promotion of local co-management actions in agreement with all stakeholders. This successfully led to the first No-Take Area (NTA) worldwide specifically designed for giant clam protection, in Tatakoto atoll in 2004 [14].

To continue contributing to existing efforts to sustainably manage French Polynesia’s giant clam fisheries, DRM can adopt a number of strategies. “Direct” management actions can be used to directly regulate fishing pressure, for instance by fixing quotas (i.e., take levels). Alternatively, managers can implement “indirect” actions, with the primary goal to improve spawning biomass by protecting individuals that are most likely to contribute to future stock size. This can be achieved using various tools (e.g., NTAs, rotationals closure, minimum size catch limits etc…). For instance, building upon the first NTA implemented in Tatakoto in 2004, Gilbert et al. [15] suggested an extended network of 9 small additional NTAs for this atoll. Another indirect strategy to reduce fishing pressure on natural stock is to promote aquaculture and spat collection [9]. Indeed, spat collection and clam farming offer a promising sustainable opportunity to supply the meat and aquarium trade, and the development of such activities was strongly endorsed by the DRM [16]. However, in order to effectively implement such activities and reliably estimate the likely impact of fishing and conservation actions on overall stock size, the DRM was missing a sensitivity analysis on the effectiveness of the different possible management strategies.

Here, we compare and quantify the long term effects of both direct and indirect management actions on the giant clam stock in two distinct lagoons of the Austral Archipelago, namely Tubuai and Raivavae. Specifically, we report on the use of a deterministic Viable Population Analysis (VPA) and spatially-explicit age-based population model that simulated the 30-year trajectory of a *Tridacna maxima* stock under different management scenarios. Under the assumption of a decreasing stock over time, we determined which scenarios could best slow down the stock decline. Given the importance to the DRM of management effectiveness and compliance on resource status, we also discuss the likelihood that the management actions will be accepted by local populations. The results, although detailed here for French Polynesia islands, provide fresh insights into giant clam management that should be of interest to a broad spectrum of Pacific Ocean Islands.

## Materials and Methods

Field methods did not require approval from any relevant body as they are harmless to giant clams and meet all applicable standards for the ethics of experimentation and research integrity.

### Study Sites and Tridacna Maxima Abundance

**Figure 1:**
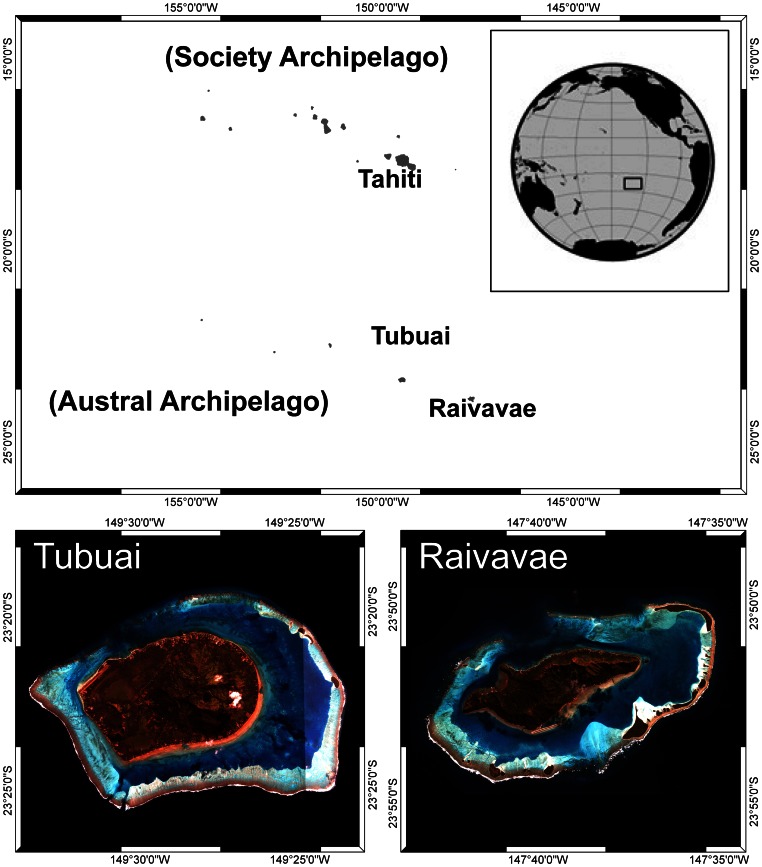
Location map of Tubuai and Raivavae (Austral Archipelago, French Polynesia).

Tubuai (23.3764 S and 149.4839 W) and Raivavae (23.8678 S and 147.6622 W) are two islands of the Austral Archipelago, in French Polynesia (Fig. 1). Their lagoons cover 90 km^2^ and 75 km^2^ [9], [17], with 2200 and 940 inhabitants respectively. Tourism is at an early stage of development and agriculture is the main commercial activity. Eighty six percent (Tubuai) and 96% (Raivavae) of all catches are destined for Tahiti’s market [18]. Jouve [18] counted between 17 and 22 fishermen at Tubuai and between 13 and 28 fishermen at Raivavae in 2010. For respectively 5 and 2 of them, giant clam fishing is their only activity and only source of benefit.

Both islands have a number of habitats suitable for giant clams. Previous studies highlighted very high biomass stocks of giant clams with estimates of 2173±232 tons for Tubuai and 469±77 tons for Raivavae [9], [17]. In 2011, The *T. maxima* fishery allowed for the collection of ∼25 and 13 tons of clam flesh fished in Tubuai and Raivavae respectively (unpublished DRM data). These are export values, and do not include local consumption.

A previous 18 month-long study conducted by DRM provided clam growth and natural mortality estimates for two sampling sites in Tubuai (Table 1) [15]. Unfortunately, these parameters were not monitored in Raivavae. In addition, *T. maxima* stock assessments were carried out twice for each island. Tubuai and Raivavae were surveyed in summer 2004 and 2005 respectively. Both islands were surveyed again in winter (June) 2010. The method used conjointly by DRM and the IRD (Institut de Recherche pour le Développement) described by Andréfouët et al. [17] was used in each island. The DRM/IRD method included the main following steps:

**Table 1:** Population dynamic parameters estimated by previous studies and used in this model.

Variable	Value	Reference
Growth	*L_t_* = 24.7×(1−*e* ^−0.0683×*t*^)	[18]
Weight growth	*W* = 0.0195×*L* ^3.0104^	[18]
Natural mortality (M)		
0–5 cm	0.11	[18]
5–8 cm	0.05	[18]
8–11 cm	0.06	[18]
11–23 cm	0.04	[18]
Age of maturity (female)	110 to 130 mm	[23]

Estimates from Gilbert [18] were assessed at Tubuai (French Polynesia), whereas estimate from Jameson [23] was assessed on Guam (Mariana archipelago).

**Figure 2:**
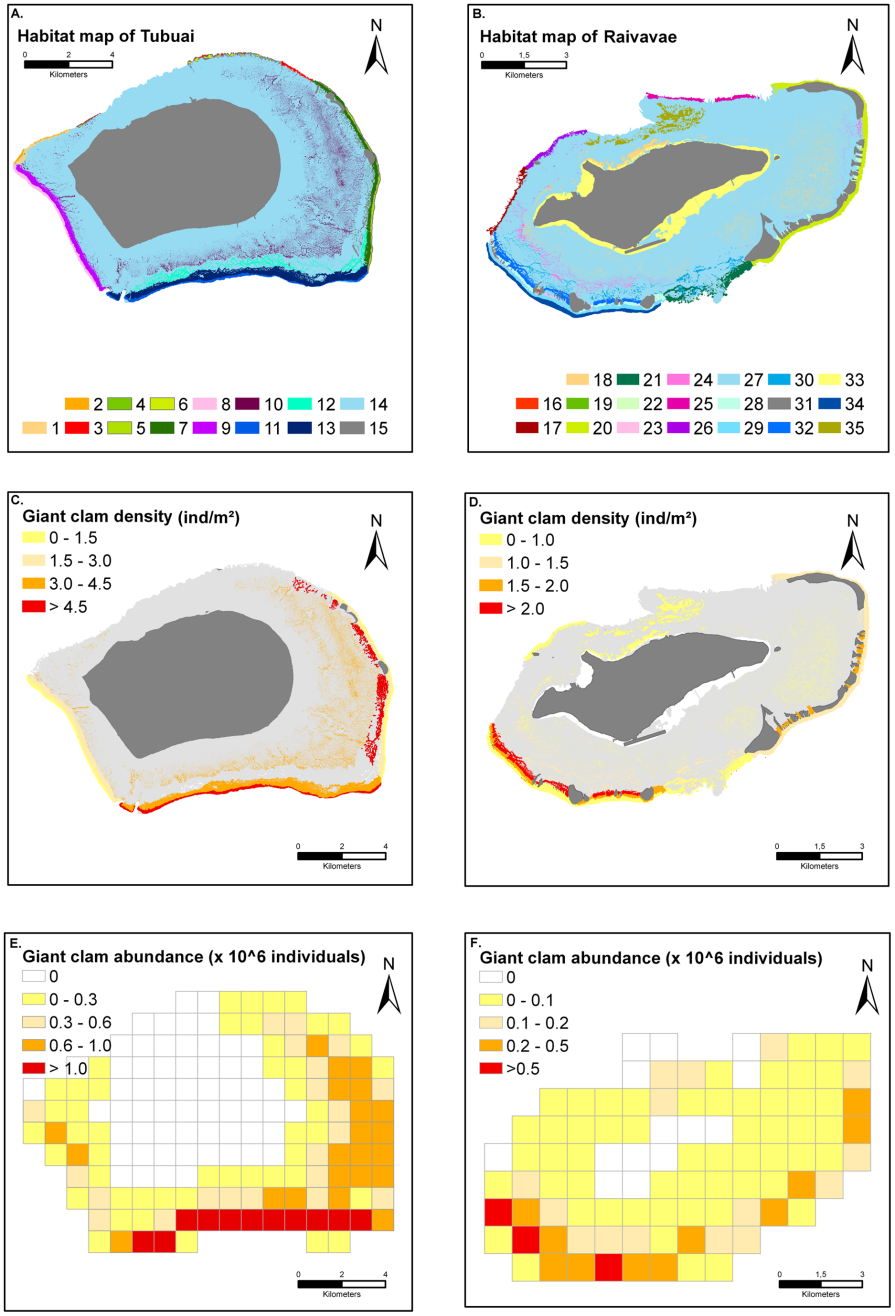
Spatial processing. For each mapped habitat (A and B), an estimate of *T. maxima* density is provided based on field surveys (C and D). For each cell, densities are then converted to absolute number of giant clam (E and F) using surface area estimates of the different habitats present in the cell. For Tubuai, habitat labels are: 1: Northwest reef flat; 2: Northwest ridge; 3: Northern ridge; 4: Eastern reef flat patches; 5: Eastern ridge; 6: Northern reef flat; 7: Eastern reef flat; 8: Western ridge; 9: Western reef flat; 10: Patch reef; 11: Southern ridge; 12: Southeast terrace; 13: Southern reef flat; 14: Lagoon; 15 Land. For Raivavae the labels are:16: Southwest reef flat; 17: Western ridge; 18: Deep patch reef; 19: Sand; 20: Motu reef flat; 21: Southern pass; 22: Hoa Motu reef flat; 23: Southwest boulder reef flat; 24: Lagoon patch reef; 25: Northern reef flat; 26: Northwest reef flat; 27: Lagoon; 28: Southwest rocky reef flat; 29: Pavement reef flat; 30: Southwest hard bottom reef flat; 31: Land; 32: Southwest back reef; 33: Fringing reef; 34: Reef ridge; 35: Northern hard bottom terrace.

- Habitat maps were created using very high resolution (2.4 m) remote sensing (Quickbird) images (Fig. 1, Fig. 2). This was done after the 2004/2005 surveys only.
- For each clam-habitat mapped (Fig. 2), 1 to 14 stations with at least 3 belt-transects per station were sampled for Tubuai and Raivavae. The number of transects was n = 301 and n = 311 for Tubuai and Raivavae, respectively. For each transect, all giant clams were counted and measured along the longest axis to yield estimates of *T. maxima* densities and size structure per habitats. In 2010, the process was reiterated for 19 and 26 of the stations previously visited (all habitats considered) for Tubuai (n = 163 transects) and Raivavae (n = 160 transects), respectively. We followed a habitat-scale stratified-random strategy. Transects were not permanently established transects and clams re-measured 6 years later were therefore not the same clams. However, transects were positioned at similar depth, sampling the same habitat, within a short distance (10 to 50 meters) of the 2004–2005 survey sites that were georeferenced with hand-held GPS. In several cases (patch reefs) the belt-transects covered a significant part of the station area, decreasing the uncertainty due to slightly different location within the same habitat, but still using a random selection within the habitat.

A detailed account of field methods can be found in Andréfouët et al. [10], Gilbert et al. [9] and Andréfouët et al. [17]. For each transect, giant clam densities were calculated as the number of clam observed divided by the surface area sampled.

### Defining Spatial Conservation Units

For each island, a grid (1×1 km cell) was generated using Geographical Information Software (GIS) ESRI ArcMap 9.2 and overlaid onto the habitat maps (Fig. 2). We assumed that each cell represented a conservation unit and could be subject to a specific management action. The cell size (1 km^2^) was a tradeoff between realistic sizes conceivable for No Take Areas in such small islands [19] and time needed for computer calculations.

The previously defined habitat maps and the giant clam density for each habitat were used to calculate giant clam abundances enclosed within each cell according to the formula:
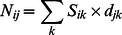
Where *N_ij_* is the number of clams of age *j* in the cell *i*, *S_ik_* the surface of habitat *k* present in cell *i*, and *d_jk_* the density of clams of age *j* estimated for habitat *k* (estimated from field surveys for the entire island). This yielded, for each cell, the stock of giant clams and its associate size structure both in 2004/2005 and 2010.

### Estimates of Future Giant Clam Stocks

Through field observation, Gilbert [20] defined a von Bertalanffy relationship between size distribution and age distribution of *T. maxima* at Tubuai (Table 1). Following this relationship, 45 age classes of one year duration could be defined (age 0 to age 44). We then used an age-based Leslie matrix [21], [22] to estimate future stocks of giant clams enclosed within each cell. We favored matrix modeling over simultaneous differential equations based models (e.g., [23]) for its simplicity, its common use in marine and terrestrial ecology [24], and the fast computer calculation. With Leslie matrices, stocks at year *t+1* can easily be calculated from the stocks of year *t* using the single equation described below.
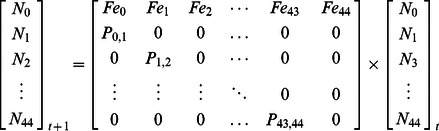



Future stocks are estimated for each conservation unit which contained suitable giant clam habitat. Therefore 102 and 84 matrices were defined for Tubuai and Raivavae respectively. We conducted VPA over a 30-year period, a good compromise between computer calculation time needed to run the various scenarios, and the time required for the entire population to be renewed by new recruits. The parameterization of Leslie matrix models consists in estimating the probability of survival from one age class *i* to another *j* (*P_i,j_*), and the number of recruits produced per clam of age *i* per time step *t* (*Fe_i_*). *P_i,j_* is a probability to survive between year *t* and *t+1* (*P_ij_* expressed in percentage per year), whereas *Fe_i_* is expressed in recruits.ind^−1^.year^−1^.

*P_i,j_* was considered as a decreasing function of total mortality (i.e., natural and fishing mortalities combined) (Table 2). The rationale is that a given cohort will progressively lose individuals over time, but at different rates according to age. For natural mortality, we used the previous estimates by Gilbert et al. [15] acquired at Tubuai Island (Table 1). Since natural mortality was measured in Tubuai only and for a relatively short period of time (18 months), we ran a sensitivity analysis to test the robustness of stock estimates to small variations in natural mortality values.

**Table 2:** Formula used to parameterize survival and fishing mortality and fecundity in the population dynamic model.

Variable	Value
Survival between ages X and X+1 (P_x,x+1_)	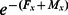
Fishing mortality at age X (Fx)	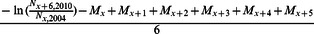
Number of recruits per clam of age X (Fe_x_)	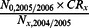

With F_x_: fishing mortality at age x; M_x_: natural mortality at age x; Fe_x_: Number of recruits produced per clam of age X; N_0,2005/2006_: the number of recruits estimated in 2005 2006; CR_x_: the contribution of age x to recruitment, calculated from Jameson [25].

For this sensitivity analysis, we first ran a “control” scenario. For this “control”, values of natural mortalities were from Gilbert et al. [15] (Table 1). Then, we ran 10 iterations, by successively decreasing and increasing values of natural mortalities from 0.01 to 0.05 units (in 0.01 increments). Note that for 11–23 cm giant clams, a decrease of 0.05 units could not be tested as the initial value of natural mortality (estimated by Gilbert et al. [15]) was only 0.04. In this later case, natural mortality was set to 0. The range of increase/decrease rates (i.e., from 0.01 to 0.05 units) was chosen to test a large panel of natural mortality. They represented between 9 and 100 percent of the initial values estimated by Gilbert et al. [15], depending on the age class considered. Finally, for each value of natural mortality tested, we analyzed if the total 2010-modeled stock falls into the 2010 field-observed stock 95% confidence interval (IC95%). The model was deemed reliable if at least 90% of the cells had their modeled stocks within their field-observed stock ± IC95%. Otherwise, the model parameterization was considered inadequate and not used for further analyses.

**Figure 3:**
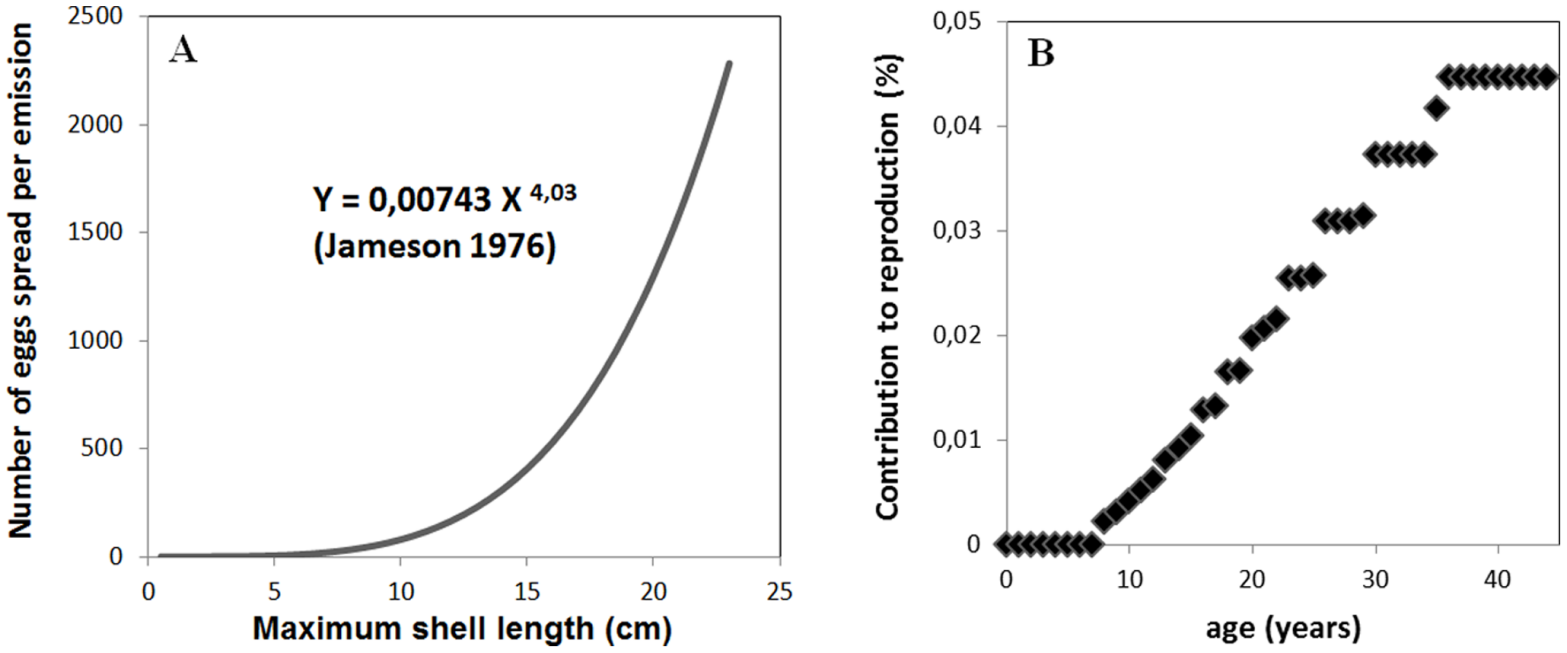
Reproductive fitness of giant clam T. maxima used in the model. A. Relationship between the numbers of eggs emitted per spawn and giant clam body size. The equation was established by Jameson [25]. The exponential relationship was used to calculate a factor for each age class' contribution to recruitment CR (B.).

For each cell, fishing mortalities were calculated as the total mortality observed between 2004/2005 and 2010 minus the natural mortality (Table 2). The number of recruits produced per clam of age *i* (*Fe_i_*) were calculated empirically using the 2004/2005 and 2010 surveys. Contribution to recruitment (%) was calculated as a function of age (Fig. 3) based on the relationship between fecundity and size given by Jameson [25]. *Fe_i_* was calculated as the number of recruits estimated in 2005/2006 (*N_0,2005/2006_*) (deduced from the number of 5 year-old clams observed in 2010 and the survivals of 0 to 5 year-old clams previously estimated) multiplied by the contribution of age class *i* to recruitment (*CR_i_*), and divided by the total number of clams of age class *i* observed in 2004/2005 (*N_i, 2004/2005_*).
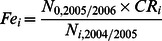



We also performed a sensitivity analysis on these estimates and we adjusted the initial values of recruits per clam of age *i* until we obtained slightly decreasing stocks of *T. maxima* (i.e., between 60 and 70% decreasing stocks during the next thirty years) as qualitatively reported by several authors (e.g., [26], [27], [28], [29], [30], [31]).

Natural and fishing mortalities are integrated into the survival probabilities (*P_ij_*) as shown in Table 2. In the model, the number of clams fished per unit of time is therefore tightly correlated with giant clam abundance. We assumed here that more available resources mean more catches. Catch is the number of clams fished, and was not linked with fishermen work intensity or catch per unit of effort (e.g., hour/days of fishing, or number of fishermen). In the case of stock decline, our model suggests that clams will be harder to find and therefore the number of clams fished will decrease. While not necessarily true for high giant clam densities found at Tubuai and Raivavae where the densities and stocks are very high, this scheme allows the direct transfer of the model to islands with limited stocks, which is the case of most South Pacific islands.

### Influence of Intra-island Population’s Connectivity

For both Tubuai and Raivavae, no quantitative data were available on intra-island connectivity. In other words, how a particular cell may be a source of recruits for another cell is unknown. No modeling results or larval census, as performed for instance by Thomas et al. [32] in Ahe Atoll in French Polynesia, were available to guide rates of exchanges for the islands in our study. Therefore, to evaluate the extent to which intra-island connectivity could affect results, two contrasting population models were established. A first model considered that the population within a conservation unit has no connections (export and import of recruits) with any other cell. Each cell was thus closed and functioned independently from the others. Hereafter, we refer to this model as the “cell structured population model”.

A second model, by contrast, considered the entire island as only one population: all cells could be a source of recruits for all others. In practice, in this population model, all cell recruits were homogeneously redistributed among all cells, as far as they included suitable habitats (whatever the proportion of suitable habitat). Hereafter, we referred to this model as the “unstructured population model”.

Clearly, these two scenarios of intra-island connectivity did not represent realistic patterns of larvae movements within each lagoon. However, these extremes helped us understand the influence of connectivity to stock changes within the lagoons. Note that these two scenarios referred to intra-island connectivity patterns only. We did not consider that recruits could be the result of larvae coming from nearby islands, as distance between islands was four times the distance of larvae dispersal observed for another giant clam species (*T. crocea*) in the Coral Triangle [33].

### Impact of Fishery Management Actions on Giant Clam Stocks

The age-based matrix model described above was used to estimate future stocks under various management scenarios. Every management action tested (described below) was applied for thirty years, starting in 2013. Their respective impacts on giant clam stocks were evaluated by monitoring the spawning biomass over time. The spawning biomass reflects a population’s resilience (i.e., ability to recover from perturbations or disturbances; see Melville-Smith [34]) and also gives a good approximation of the stock size allowed for commercialization (i.e., biomass from clams >12 cm given French Polynesian laws).

We first considered a direct management action, by testing various limiting quotas for the yearly fished biomass. We also considered four indirect management scenarios, namely No-Take Areas (NTAs), rotating closures, minimum clam size limits, and aquaculture/restocking. We did not model a combination of scenarios (e.g., quotas+rotating closure).

For NTA scenarios, we systematically searched which conservation unit would increase the overall island stock if protected. For Tubuai, we also considered the network of 3 NTAs proposed by Gilbert [15] and tested 3 sizes for NTAs (2 km^2^, 4 km^2^, and 8 km^2^). Three scenarios of rotating closures were considered, protecting 25%, 33% and 50% of the lagoon area each year. In each case, protected cells were all immediately adjacent. When partially closing access to the resource (NTAs or rotating closure), we assumed that catches (i.e., number of clam fished) shifted towards the closest accessible cells.

For size-limits, we tested the effect of increasing the current minimum size limit from 12 cm to 16, 18, and 20 cm, and tested these same values as maximum size limit. In these scenarios, the catch was transferred to all size classes open to fishing.

Finally, we modeled various amounts of restocking (from 100 to 10 000 seven years old individuals in each conservation unit), assuming these clams would come from spat collection and growth in nursery tanks protected from predators.

All scenarios and parameterizations are summarized in Table 3.

**Table 3:** Summary of all strategies of fishery management and all scenarios tested.

Strategy	Scenario tested
*No-Take-Areas*	1 NTA (100 locations tested)
	3 NTAs of 2 km^2^ (1 location tested)
	3 NTAs of 4 km^2^ (1 location tested)
	3 NTAs of 8 km^2^ (1 location tested)
*Rotating closures*	2 Rotating closures (5 locations tested)
	3 Rotating closures (5 locations tested)
	4 Rotating closures (5 locations tested)
*Size limits for catch*	>16 cm
	>18 cm
	>20 cm
	>12 cm and <16 cm
	>12 cm and <18 cm
	>12 cm and <20 cm
	>16 cm and <18 cm
	>16 cm and <20 cm
	>18 cm and <20 cm
*Restocking*	100 ind/cell/year
	1 000 ind/cell/year
	5 000 ind/cell/year
	10 000 ind/cell/year
*Quotas*	45 tons/year
	50 tons/year
	55 tons/year
	70 tons/year

## Results

### *T. maxima* Stock Abundance Assessment

For both islands, giant clam densities varied depending on habitats’ geomorphological and geographic characteristics (Fig. 2).

In Tubuai Island, the southern ridge and reef flat displayed very high densities (3.7 and 5.8 ind.m^−2^ in 2004, and 4.6 and 3.8 ind.m^−2^ in 2010 respectively). The eastern and western ridge and reef flat populations were less dense (from 0.2 to 2.2 ind.m^−2^ in 2004 and from 1.1 to 2.0 ind.m^−2^ in 2010). The eastern half of the lagoon contained many patch reefs with very high giant clam densities. The northern part was by contrast less densely populated.

Similar patterns were observed in Raivavae. The southwestern reef flat’s hoa (a shallow channel separating two reef islands [35]) displayed the greatest densities of giant clams (up to 7.4 ind.m^−2^ in 2005 and 6.2 ind.m^−2^ in 2010), whereas the northern reef flat harbored less than 0.17 ind.m^−2^ in 2005 and 0.35 ind.m^−2^ in 2010. Just like in Tubuai, clams were found in high densities on many patch reefs throughout the lagoon. The northern deep hard bottom substrate sheltered high densities of large giant clams only (>18 cm), with little fishing activity reported likely due to the presence of ciguatoxic clams in this area [36].

**Figure 4:**
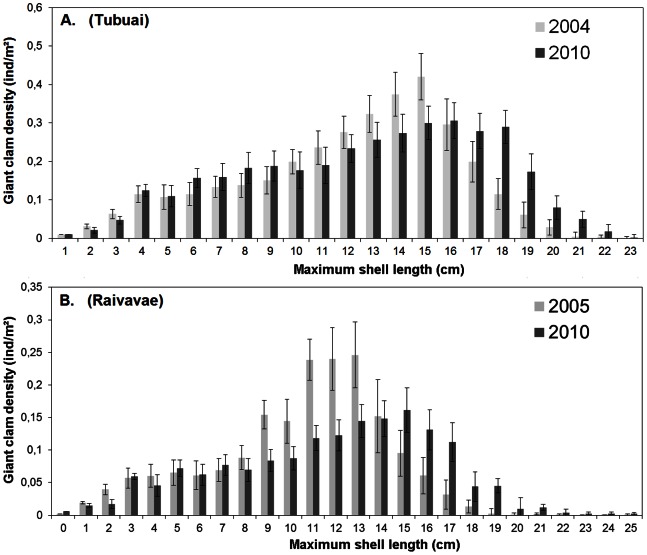
Size structure of *T.* *maxima*. A. represents the evolution of *T. maxima* densities for each size class between 2004 and 2010 for Tubuai, and B. for years 2005–2010 for Raivavae.

For both Tubuai and Raivavae islands, the population trajectory between the 2004/2005 and the 2010 surveys (Fig. 4), showed increasing densities of large giant clams (>18 cm) and a slight decrease in overall densities. Large clams, however, appeared less abundant in 2010 than what the evolution of the 2004 size structures would suggest. This likely reflects the impacts of fishing activities over the last few years with fishermen preferentially targeting large clams (personal observations).

### Modeling Population Dynamic of *T. maxima*

**Figure 5:**
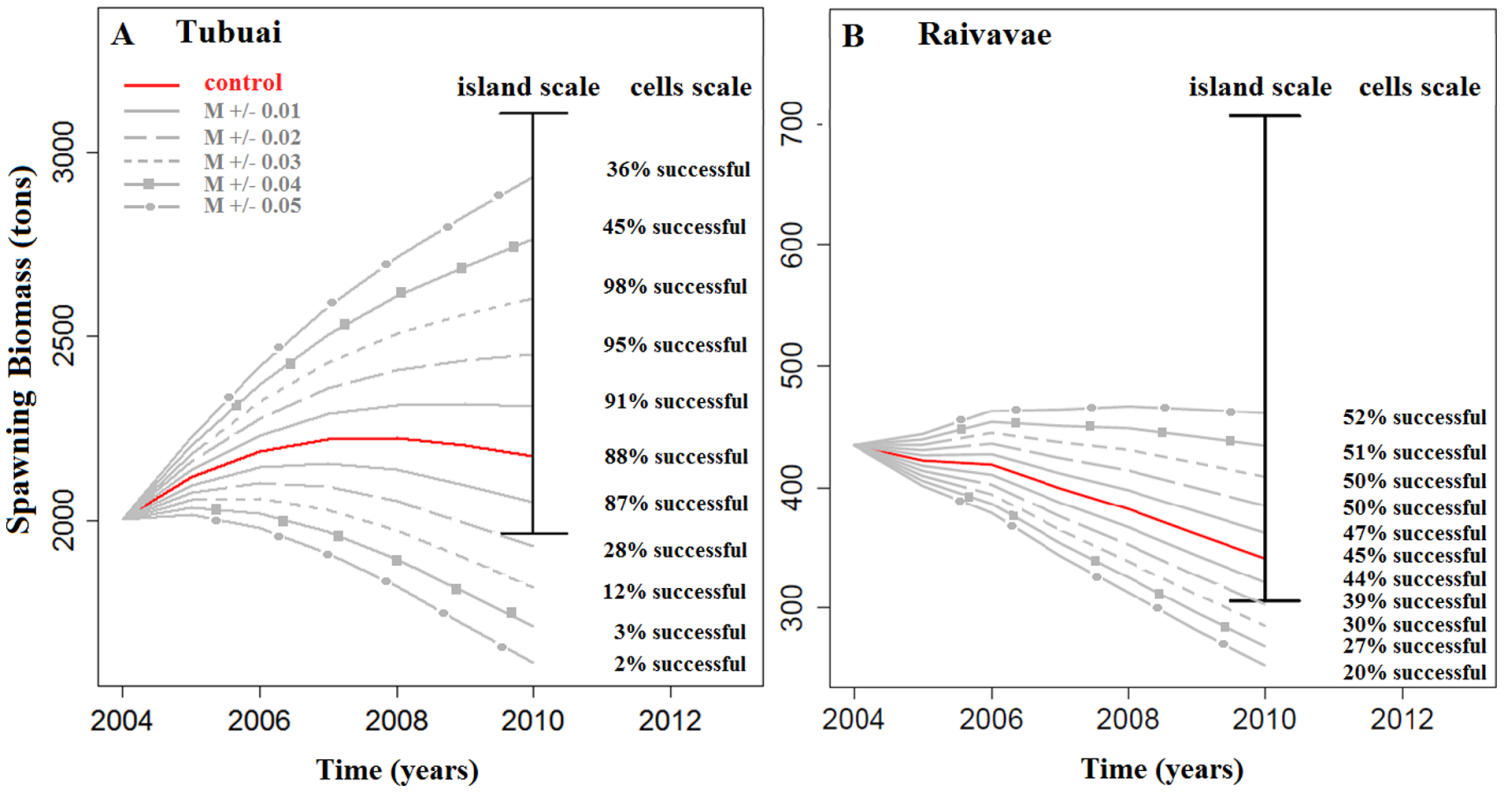
Projection of future stock status in tons of spawning biomass. Projections for Tubuai Island (A) and Raivavae Island (B) as estimated by the model and sensibility analysis according to various estimates around the natural mortality (M) arrived at by Gilbert [15]. The reliability of the model is evaluated by two metrics. First, at island scale, the model is considered reliable if the 2010-modeled stock falls within the field-observed stock IC95%. Then, at cell scale, model reliability is evaluated by the proportion of cells for which 2010-modeled stock falls into the field-observed stock ± IC95% (indicated as “successful” on the figure).

For the Tubuai Island model, the estimated mortality rates were in good agreement with field observations from 2004 and 2010. When testing the sensitivity of the output to natural mortality rates, we noticed that when decreasing the value initially estimated by Gilbert et al. [15] by 0.01 units, a correct stock was estimated for 91% of the cells in 2010 (Fig. 5). For Raivavae, model results were less in agreement with observations. Decreasing Gilbert et al. [15]’s natural mortality by up to 0.05 units was still insufficient to yield an estimate in agreement with 2010 observations.

**Figure 6:**
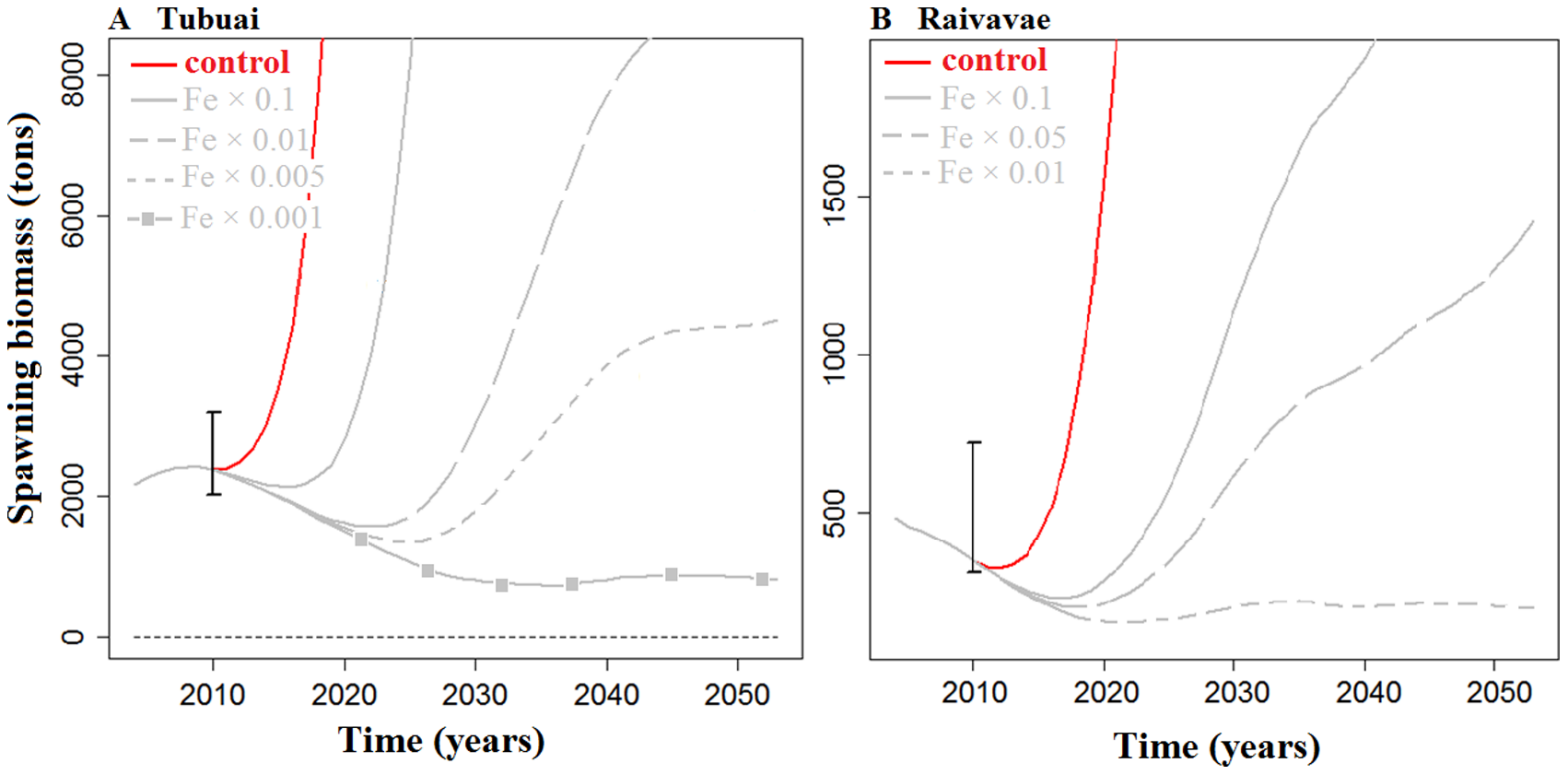
Projection of future stock status in tons of spawning biomass for Tubuai Island (A) and Raivavae Island (B) estimated by the model, with sensitivity analysis for the number of recruits produced per clam (Fe) estimated from field measurements. Fe had to be decreased by a factor of 1000 at Tubuai and by 100 at Raivavae to obtain realistic trajectory results (i.e., a 70% decrease of stocks over the next thirty years).

For both islands, when using field-based estimates of recruits per clam of age *i (Fe_i_)*, stocks increased rapidly reaching unrealistic levels after only a few years (Fig. 6). In other words, if recruitment was annually at the same level as in 2004, the number of clams would increase exponentially. This exponential growth is not attributed solely to the recruitment levels, but to the inherent way the Leslie matrix is constructed, including growth and survival. However, as recruitment measurements are far more uncertain than survival and growth measurements, we rather adjusted the recruitment rate. We had to decrease this initial recruitment rate (*Fe_i_*) by a factor of a 1000 for Tubuai and 100 for Raivavae, to forecast realistic estimates of stocks over the next thirty years.

Given that model results were more realistic for Tubuai, probably because the population dynamic parameters used in the model were measured in situ in Tubuai, we focus the following results section on Tubuai Island only. For this island, the ranges of values used to build Leslie matrices are provided as (Fig. S1).

### Effectiveness of Fishery Management Actions

**Figure 7:**
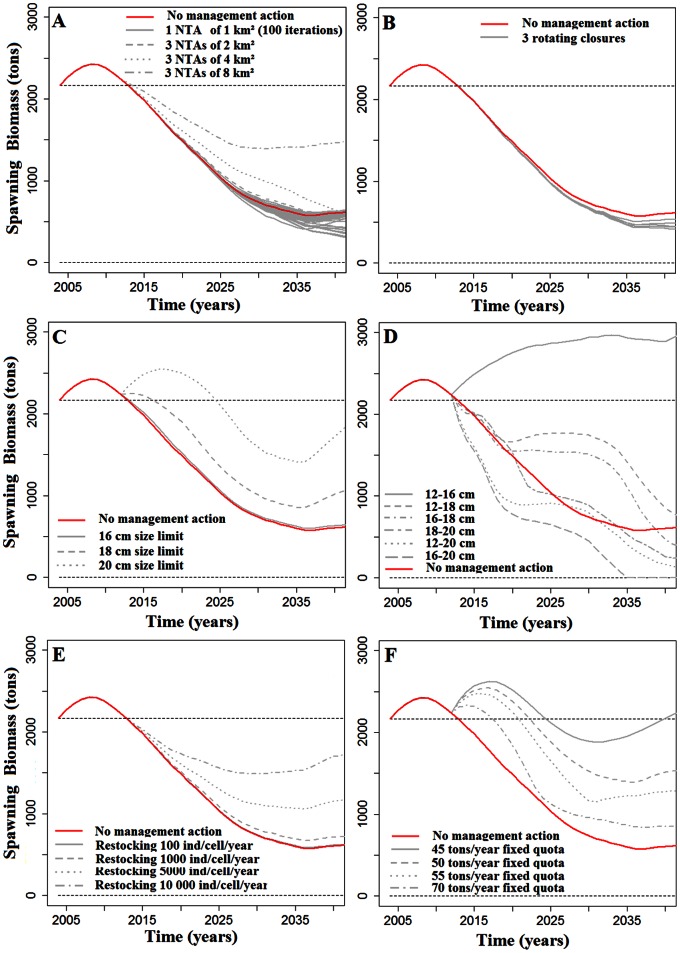
Projection of future stocks in tons spawning biomass for Tubuai Island estimated by the unstructured population model (i.e., all cell recruits were homogeneously redistributed among all cells, as far as they included suitable habitats) under various fishery management scenarios. Scenarios presented here are: A. implementation of one No-Take-Area of 1 km×1 km, and a network of three NTAs of 2 km^2^, 4 km^2^, and 8 km^2^, B. three rotating closures, each protecting 33% of the lagoon, C. Minimum size limit fixed at 16, 18 or 20 cm, D. Minimum and maximum body size limit for catch set at 16, 18 or 20 cm, E. Yearly restocking of 100, 1 000, 5 000, and 10 000 giant clams per cell, and F. Fixed quotas of 45, 50, 55 and 70 tons of clam flesh fished per year.

The effect of the various management strategies on the Tubuai spawning biomass of giant clam *Tridacna maxima* is presented in Fig. 7. The figure shows the outputs under the assumption that all cells are connected by larval dispersal (unstructured population model). Model outputs with the assumption that all cells are closed (cell structured population model) are provided as (Fig. S2).

Surprisingly, the two models of intra-island connectivity yielded very similar results on management effectiveness. We observed small differences for area-based scenarios (i.e., NTA and rotational closure), but these were negligible when compared to the overall island stock.

For area-based management, overall, only very few scenarios were effective (Fig. 7A). Indeed, only closing off a few specific areas to fishing had positive effects on stock abundance. Scenarios also showed that closing cells actually slightly negatively impacted the regional stock for 86% of cells. A network of three 8 km^2^-NTAs could, however, slow down the decline of stocks for the unstructured population model. Surprisingly, protecting wider areas, through rotating closures or wide NTAs for the cell structure population model led to dramatic negative effects on overall stock size.

Restocking was efficient only when a very large number of giant clams were reintroduced (Fig. 7E). With no other management action but restocking, a reintroduction of at least 5,000 individuals per cell and per year is needed to slow stock decline by 50% when compared with stock evolution without any management actions.

Banning giant clam fishing for specific size classes had various outcomes. Increasing the minimum size limit from 12 cm to 16 cm had an insignificant effect on the stock, but increasing it to 18 cm and 20 cm increased the stock by 86% and 230% respectively in the unstructured population model (Fig. 7C). Limiting fishing between 12 and 16 cm (i.e., maximum size limit) increased stocks over time, but the stock plummeted with other size restrictions (Fig. 7D).

Limiting giant clam harvest through fixed quotas appeared to be the most efficient action to sustain the resource (Fig. 7F). Our model suggested that using quotas to decrease the current catch two fold could stop the estimated decrease of stocks over the next thirty years.

## Discussion

To the best of our knowledge, this study is the first spatially explicit model applied to forecast the sustainable harvest and management of giant clams. We discuss hereafter the weaknesses and strengths of our approach, as well as the relevance of our results to giant clam management in Tubuai and Raivavae and elsewhere in the South Pacific region.

### Strengths and Limitations of the Population Model

The main strength of the model is that it provides a spatial view of the resource and of the relative effects of different management actions on stock size and thus livelihood opportunities for local communities. Interest for spatial modeling has risen in the past decade and several spatially explicit models have recently been used to help management decisions (see [37] for an exhaustive review). For giant clams, several models have been recently developed. For instance, Gilbert et al. [15] used yield per recruit and spawning biomass per recruit to estimate fishing impact on *T. maxima* stocks of three islands in French Polynesia. In the same study, the effects of rotating closures and quotas were tested at island scale, but without any spatial information. Yau [38] also used an Integral Projection Model to define an optimized size limit for the *T. maxima* fishery on Moorea island (French Polynesia) but also without using a spatial model.

The availability of a spatial model, constrained initially by habitat distribution and clam density per habitat type, refines considerably the level of knowledge available to managers. Stocks are fished at a very local scale (grid cells of 1 km×1 km here) and management actions and their impacts can be analyzed at the same level, increasing the relevance of a management plan for local inhabitants.

Nevertheless, some limitations should be pointed out. First, we considered natural mortality to be uniform throughout the entire lagoon at Tubuai and Raivavae Island. This was obviously a weak assumption for Raivavae since our modeled stock size estimates were outside the confidence range of uncertainty estimated from our field data (Fig. 5). For future work, we recommend to monitor spatially these parameters at adequate time intervals. One relevant approach would be to stratify future demographic sampling (survival, growth and recruitment) by habitat types. Indeed, habitat typology has been established to map giant clam stocks using remote sensing for several atoll/island of French Polynesia (e.g. Tatakoto, Reao, Tubuai, Raivavae). The different habitats provide various environmental conditions (temperature, depth, hydrodynamic, turbidity), which may influence giant clam biology and demographic processes [39], [40], [41], [42]. Revisiting sites frequently (we suggest twice a year) should help in systematically quantifying the variability in recruitment [43], [44] and the occurrences of mortality events [12], [13].

Second, to be relevant for most low-stock Pacific islands, we modeled the fishing mortality as a constant rate over time. This implied a decreasing number of fished clams when stocks decrease and *vice versa*. While this assumption is justified when a resource is scarce (e.g. [45], [46]), this is not necessarily the case for several islands and atolls of French Polynesia where giant clams are highly abundant [10]. Third, the correlation between survival and stocks used in the model is not density-dependent *sensu stricto* as survival probabilities are constant values and are independent of the stock itself or the observed densities. In most population dynamics, density dependence of survival is effective when resources are limited and must be shared between individuals (extensive literature is available since Verhulst [47]). Such models thus consider a negative correlation between survival and density. The higher the density, the lower the survival (in particular for juveniles). This is not necessarily true for giant clams as their resources are mostly provided by their symbiont photosynthetic zooxanthellae and because intra-species competition is low (but see Hamner [48]). *A contrario*, reproduction success may be positively density-dependent for giant clams, due to various physical and chemical signals that trigger simultaneous spawning [49]. However, the lack of quantitative data on this phenomenon precluded its accurate modeling and we did not explicitly consider density-dependence in our model. In practice, density dependence of reproduction success is a very difficult parameter to estimate in the field. One may test various scenarios of density dependence, but the added complexity and increased computing time may not be justified until a realistic range of values is targeted.

Fourth, contribution to recruitment (*CR*) was calculated from a relationship between fecundity and size. This means that we considered that gametes/larvae from various clam size classes have similar probabilities of fertilization, survival, and fixation. For older age classes, the relationship appeared stair stepped as one size class could correspond to several age classes.

Fifth, we did not consider inter-island connectivity in our model. Inter-island connectivity of giant clams has been addressed in several studies. DeBoer et al. [33] found very low dispersal distances (25–50 km on average) for Tridacnidae in the Coral Triangle in Asia. In French Polynesia, Laurent et al., [50] demonstrated high structuration between archipelagos only, but using allozyme loci (n = 10). They could, however, establish a relation between connectivity and geographical distance. Considering the distance between our focus islands (200 km), and oceanic currents, the number of recruits coming from other islands must be negligible compared to local recruits and their impact on the 30 years estimates of stocks unnoticeable. More extensive studies of larvae connectivity would help confirm this assumption.

Finally, broadcasted recruits per clam of size *i* was set to match a realistic decrease in the number of individuals as observed in other islands. A slow decrease has been set as a reference because slow depletions have been reported in French Polynesia [51] and in many tropical Indo-Pacific islands [52] due to overexploitation [53]. However, very few long term surveys have been performed and to the best of our knowledge, stock declines have only been described qualitatively for Pacific Ocean islands. Giant clam stocks seemed to have decreased two fold in the Red Sea [54], [55], and massive mortalities have been reported in atolls of French Polynesia (e.g., [56], [13]). However, no data on Pacific Ocean islands could help adjust the slope of stock decline used in our study (between 60% and 70% decrease in thirty years for Tubuai and Raivavae respectively). Setting Fe_i_ (i.e. recruits per clam of age *i*) to match a foretold decline in stocks remains a fairly qualitative parameterization, but this did not preclude a comparison among the different management scenarios outputs.

In short, our giant clam population model suffers from a lack of spatial and temporal data on mortality, both natural and fishing, recruitment, and larval connectivity. The parameterization we have used is realistic but lacked fine tuning of important population dynamics parameters to accurately model stock evolution over the next 30 years for a given island. The results achieved for Raivavae and Tubuai suggest that local parameters of population dynamics are needed to reach a good agreement between field observations and model outputs (Fig. 5, Fig. 6). Indeed, using the natural mortality measured in Tubuai apparently could not provide reliable stock estimates for Raivavae despite the proximity and the similar size structure between these two islands [9]. The sensitivity analysis helped identify in which direction a model parameter needs to be fine-tuned to match local field data. But, clearly, using our spatial model in new sites with very different environments and clam population structures (e.g., Tuamotu atolls instead of Austral Islands) will require the collection of *in situ* data to adequately parameterize the model and strengthen reliability in forecasts. In addition, knowledge of intra-lagoon connectivity, larval dispersal and currents would be useful [32].

### The Recruitment Factor

Considering recruitment, our model suggests a high temporal variability. Indeed, *T. maxima* population size exploded when we considered a stable number of recruits per clam of age i (*Fe_i_*) matching the 2004–2005 observations (Fig. 6). The 2004–2005 observed recruitment may have been a particularly good episode, but we lack monitoring data to measure how far from an average year this may have been. Numerous studies have shown the high variability of recruitment for benthic invertebrates and its consequences on population structures and stocks. Sainsbury [43] used a sized based Leslie matrix model to show that only a succession of short periods of high recruitment followed by longer periods of low recruitment could explain the observed size structure of the shellfish *Haliotis iris* in New Zealand. For giant clams, recruitment has also been described as erratic in hydrodynamically open locations [44], although recruitment could be regular and steady in closed lagoons like the Tuamotu lagoons. Massive mortality events, followed by low recruitments years, also highlight the strong variation of natural mortality in connection with weather events [12], [13].

Recruitment is dependent on a wide array of processes including climate and weather forcing, local hydrodynamics immediately after spawning periods, reproduction potential (according to sizes and aggregations, which can be modified by fishing), substrate suitability [57], larval dispersal [23], [58], natural predation on larvae and adults [23], [58]; and competition with other species [23], [58]. Perfect analytical understanding of all these parameters and processes is unlikely to be achieved for most sites without extensive (and prohibitive) fieldwork. Nevertheless, empirical understanding can come with regular monitoring of the population using a habitat-based spatial design as discussed above. In practice, at least in French Polynesia, sampling frequency is always a trade-off between precision required (ideally a bi-annual sampling program) and funding available for field sampling in remote islands.

The observed high spatial and temporal variability of population dynamics parameters may force managers to collect new data frequently, and possibly often adjust management strategies accordingly. Indeed, from a scientific stock management perspective a particular quota should be updated regularly according to the most recent stock estimates, and especially after massive mortalities [13]. Yet, this may represent socio-political challenges of its own. Future challenges in the actual context of climate change will be to determine the vulnerabilities of a particular site to the occurrence of high mortality events, or changes in recruitment patterns, due to thermal and hydrodynamic stress and variations. In fact, what is actually currently unknown and poorly documented in the literature is the frequency by which data should be updated to keep management actions efficient over time.

### The Poor Efficiency of No Take Areas (NTAs)

NTAs have received increasing attention over the past decades as a useful tool for the management of impacted or soon-to-be-impacted resources [59]. Many studies highlight their positive effects [60], such as the increasing body size of fished resources [61], [62], [63]. NTAs’ benefits are often species-dependent. For short-lived species, NTAs can quickly lead to complete stock recovery with noticeable positive effects on stocks both inside and outside NTAs, whereas for sessile and longer-lived species, positive NTA effects may be measured after decades only [64], [65]. The density dependence of giant clam’s ability to reproduce may help the recovery of a protected stock, but whether the effects can be positive both within and outside the NTAs is likely case-dependent. Halpern and Warner [66] for example suggested that a local NTA could be of interest for an entire regional stock. Indeed, “spill-over effects” can bring individuals of mobile species from inside the NTA toward the open areas, enhancing biomass outside the NTA, and possibly enhancing catch statistics [67]. In the case of non-mobile adults like clams, a spill-over effect is materialized by enhanced dispersal of fertilized eggs and larvae released from the NTA [68]. However, the differences between our unstructured population model and cell structured population model do not suggest a high sensitivity to spill-over of gametes at the island scale.

Here, we found that closing local areas to fishing will actually decrease the overall stock of the island. Several authors have reached similar conclusions [69], [70], [71]. This can be explained by fishing effort redistribution targeting highly fecund areas. This phenomenon is well-known and discussed in the Marine Protected Area literature, usually discussed under “edge-effects” and “displacement of fishing effort” [72], [73]. In our model, closing areas to fishing meant a reallocation of effort and thus increased number of clams fished in neighboring areas. In fact, for 86% of our conservation units, closing the area to fishing increased the stock inside the NTA, but depleted neighboring stocks until extinction. We observed this for both the unstructured population model and cell structured population model. Only a network of three NTAs of 8 km^2^ (i.e., 24 km^2^ protected) could slow down the decrease of stocks for the unstructured population model. This represented a protection of 27% of the lagoon, which seems unrealistic for compliance reason. For this scenario, however, we kept the same design catch reallocation as earlier (i.e., to directly adjacent cells), which could overestimate stocks if the reallocation of fished clams exceeded the directly adjacent cells stock. This conclusion opens new directions for socio-economic surveys. Specifically, it could be very useful for managers to understand how and where clams would be harvested from if a specific NTA was to be established.

### Relative Merits of Management Actions

Managing quotas as a direct management action is a promising tool to increase stocks effectively. Our model suggests that decreasing catches two-fold using fixed quotas could increase the number of *T. maxima* individuals compared to current stock abundance. According to the recent estimate of ∼25 t of clam flesh fished at Tubuai in 2011, we therefore suggest that a fixed quota of 10–15 t.y^−1^ would increase stocks over the 30 next years.

Among indirect management actions, the most efficient tool according to our model is to increase the minimum shell size limit. Increasing the current 12 cm minimum size limit would allow giant clams to reproduce several times before being fished, as sexual maturity (male and female) is assumed to occur at 11 cm [25]. The efficiency of such method has been described widely [74], [75], [76], [77]. It was used for scallops [78] for example and Yau [38] in Moorea island (French Polynesia) used a non-spatial model to show that a size-limit of 13.5 cm for giant clams could locally ensure the best compromises between catches and abundance, independently of recruitment rates. Note that the positive effect of a minimum size limit on stocks may be underestimated here since clam sales are expressed in weight, whereas redistribution of fishing effort was expressed in clam abundance in our model. This mean that, for the same amount of commercial weight sold, fewer individuals are required if caught clams are of greater size. For the same reasons, the positive effect of maximum size limit may be overestimated.

The local restocking of individuals would be of insignificant effectiveness unless several hundreds of thousands of giant clams were reintroduced over several years (Fig. 7). Such large scale restocking may be a future possibility considering the development of aquaculture and spat collection in French Polynesia, but it seems insufficient unless massive efforts are coordinated. Restocking could nevertheless be used to increase awareness and interest in giant clam management among the local population, fishermen, and aquaculturists.

Direct management actions (such as quotas per island) limit the overall catches, but do not explicitly control the spatial repartition of fishing effort. As such, reduction of catches does not necessarily ensure sustainability if the overall (allowed) fished biomass affects a size class or a location of critical importance to the stock. *A contrario*, indirect management actions alone (such as NTAs) control fishing repartition, but without biomass limitation. Direct and indirect management actions are therefore complementary. To complement the actual size limit of 12 cm, based on our results we would recommend a reduction in total catch until future stocks are sustainable (i.e. until stocks are not decreasing any more). In practice, such a reduction in catch could first be legally endorsed (i.e., by setting quotas) until aquaculture operations are developed enough to replace wild harvests.

### Giant Clam Fishery Management for Islanders: at what Scales and for which Strategies?

Fishery management often involves complex interactions at a number of different scales, to answer national, international, and local needs. As a result, some management actions are widely applied and valid for all of French Polynesia, whereas others are specific to an island’s needs. Within the remit of the latter, Gilbert et al. [15] suggested an adaptive co-management approach. In such an approach, a committee gathers all stakeholders (i.e., fishermen, managers, scientists, and buyers), who should meet regularly to 1) establish a data gathering/fishery monitoring plan performed by scientists or at least in close relation with them; 2) evaluate the new status of the resource; and 3) decide jointly on the best way to adapt management strategies accordingly [79]. It helps for fishery regulations to be more easily understood, accepted, and respected by all stakeholders. Co-management strategies are already in place in some atolls (e.g., Tatakoto) and have successfully led to management actions [14]. In the literature, other examples of co-management are documented, providing both successful and unsuccessful results [63], [80], [81].

Traditionally, Polynesian islanders used to implement rotating closures (locally called “Rahui”) and NTAs (“Tabu”) to manage their own resources. As such, rotating closures and NTAs are generally fairly well understood and accepted by inhabitants. Official and legal NTAs have already been implemented by the DRM in several islands, with nine additional NTAs planned for Tatakoto lagoon, and three for Tubuai [15]. However, based on our results, it would appear useful and critical to evaluate the efficiency of NTAs in place, in particular the extent to which they benefit giant clam stocks. The 12 cm minimum size limit, while not as traditional as NTA, has been fixed for all of French Polynesia since 1988, and is now well rooted in the inhabitant’s practices. Modifying this 12 cm limit may, however, be confusing. A better approach seems to be quota-based management. Our model suggests that a 12–15 tons harvest limit should increase stocks over 30 years for Tubuai – except for any unforeseen mortality events. In practice, only exportation to Tahiti’s market can be verified by a controller present at each plane and boat departure. Exportations represent 86 to 96% of fishing biomass for Tubuai and Raivavae respectively [18], and controlling them is a good way to limit fishing for complementary income without affecting subsistence fishing. Controls can be done before exportation (i.e., directly at the island/atoll), or after exportation (i.e., at Tahiti). We recommend to implement a control before exportation to keep all stakeholders involved in the process of resource management. Although this may be seen as a significant effort, one should remember that most of French Polynesia’s remote islands have only one or two planes weekly at best, and boat rotations twice per month, and sometimes even less. Thus, the amount of time devoted to shipment control is not overwhelming.

Giant clam fishery management faces two main challenges in the short term. On the one hand, we encourage scientists to adapt and implement the model described here to new lagoons, new environments (e.g., closed atolls, open atolls) and with additional forcing events (e.g., high mortality events). This generalization would confirm the robustness of quota-based management effectiveness. On the other hand, managers also need to find, for each lagoon, the best compromise between effectiveness of management actions and their compliance by all stakeholders.

## Supporting Information

Figure S1 **Range of values used for Tubuai and for each component of the Leslie Matrix used in the model, without any management action.** For spatially dependent parameters, the minimum and maximum values (separated by “-“) observed among cells are indicated. The number of recruits produced per clam was considered spatially dependent and increased exponentially with age but only clams older than 8 years were considered mature. Fishing mortality occurs only for clams holder than 9 years, and is spatially dependent. For clams younger than 9 years, only natural mortality is considered, and was not spatially dependent.
(TIF)

Figure S2 **Projection of future stocks for Tubuai in tons of spawning biomass estimated by the model under various scenarios of management.** No connectivity is considered between cells (cell structured population model). Scenarios presented here are A. implementation of one No-Take-Area of 1 km×1 km, and a network of three NTAs of 2 km^2^, 4 km^2^, and 8 km^2^, B. three rotating closures, each protecting 33% of the lagoon, C. Minimum body size limit for catch fixed at 16, 18 or 20 cm, D. Minimum and maximum body size limit for catch at 16, 18 or 20 cm, E. yearly restocking of 100, 1 000, 5 000, and 10 000 giant clams per cell, and F. Fixed quotas of 45, 50, 55 and 70 tons of clam flesh fished per year.
(TIF)
