# Supplementary figures and images for: Best Management Strategies for Sustainable Giant Clam Fishery in French Polynesia Islands: Answers from a Spatial Modeling Approach
Source: PLoS One. 2013 May 28;8(5):e64641. doi: 10.1371/journal.pone.0064641 (PMC3665777; doi:10.1371/journal.pone.0064641)

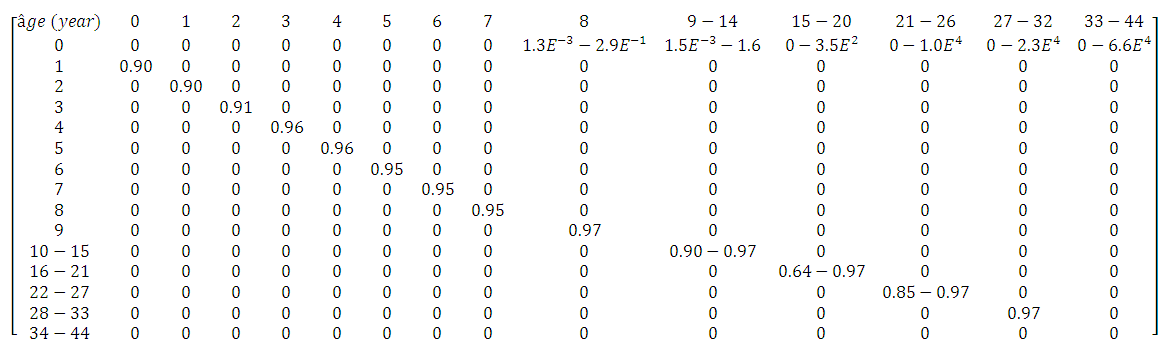

Supplement: Figure S1 — Range of values used for Tubuai and for each component of the Leslie Matrix used in the model, without any management action. For spatially dependent parameters, the minimum and maximum values (separated by “-“) observed among cells are indicated. The number of recruits produced per clam was considered spatially dependent and increased exponentially with age but only clams older than 8 years were considered mature. Fishing mortality occurs only for clams holder than 9 years, and is spatially dependent. For clams younger than 9 years, only natural mortality is considered, and was not spatially dependent. (TIF) [file pone.0064641.s001.tif]

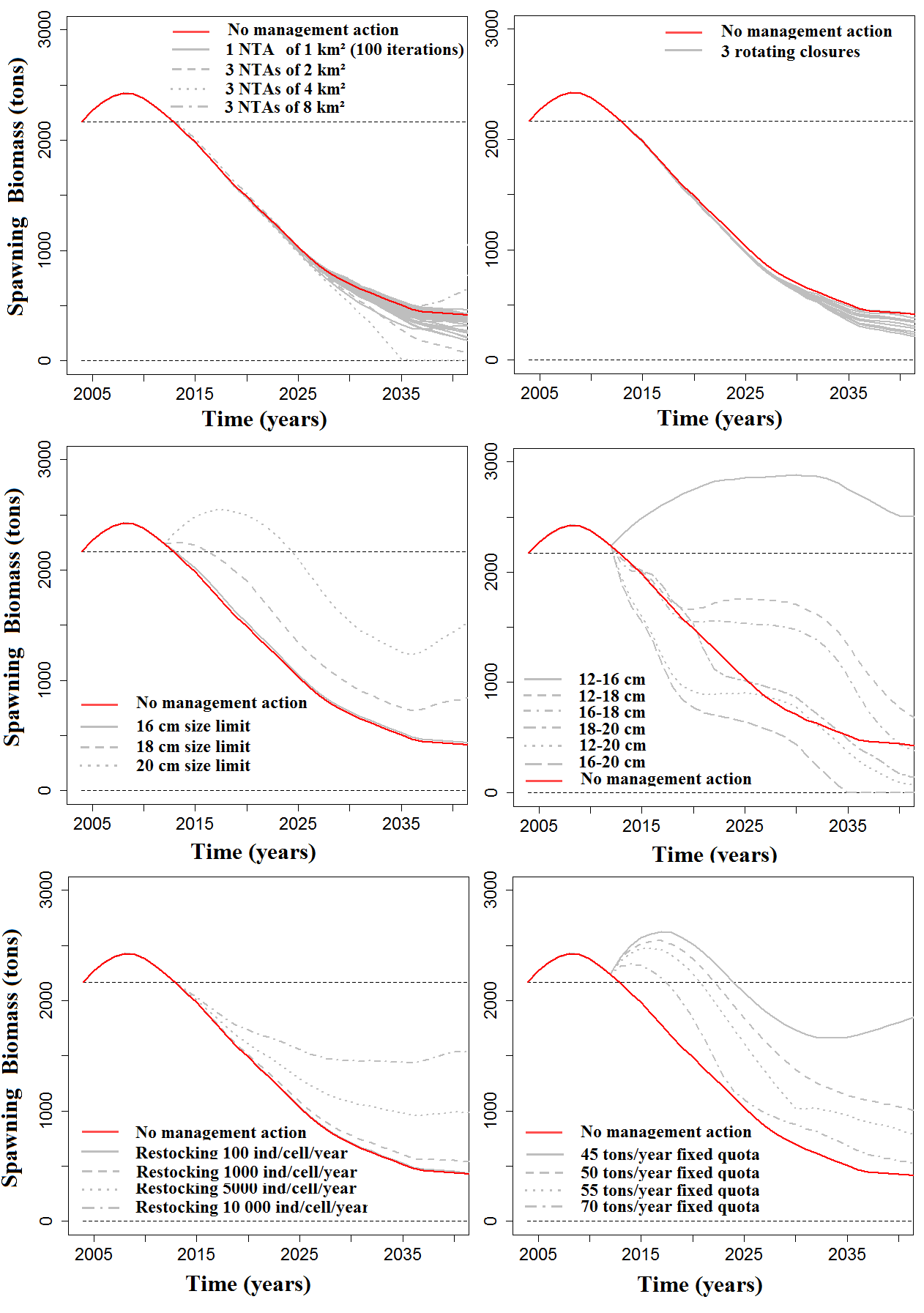

Supplement: Figure S2 — Projection of future stocks for Tubuai in tons of spawning biomass estimated by the model under various scenarios of management. No connectivity is considered between cells (cell structured population model). Scenarios presented here are A. implementation of one No-Take-Area of 1 km×1 km, and a network of three NTAs of 2 km2, 4 km2, and 8 km2, B. three rotating closures, each protecting 33% of the lagoon, C. Minimum body size limit for catch fixed at 16, 18 or 20 cm, D. Minimum and maximum body size limit for catch at 16, 18 or 20 cm, E. yearly restocking of 100, 1 000, 5 000, and 10 000 giant clams per cell, and F. Fixed quotas of 45, 50, 55 and 70 tons of clam flesh fished per year. (TIF) [file pone.0064641.s002.tif]
